# Effects of N Application Rates and Ratios on Photosynthetic Characteristics and Dry Matter Production of Different Rice Varieties

**DOI:** 10.3390/plants15142217

**Published:** 2026-07-21

**Authors:** Jinghong Ji, Jingfang Xue, Xingzhu Ma, Yongsheng Cai, Shuangquan Liu, Xiaoyu Hao, Yu Zheng, Yue Zhao, Yuqi Xia, Shuqiang Chen

**Affiliations:** 1 Heilongjiang Academy of Agricultural Sciences, Harbin 150086, China; 2 Scientific Observation and Research Station of Soil Quality, Key Laboratory of Black Soil Protection and Utilization, Ministry of Agriculture and Rural Areas, Harbin 150086, China; 3 Jiamusi Field Comprehensive Scientific Observation and Research Station, Ministry of Agriculture and Rural Affairs, Jiamusi 154026, China; 4 Scientific Observation and Experimental Station for Cold Damage of Japonica Rice in Cold Regions, Ministry of Agriculture and Rural Affairs, Jiamusi 154026, China; 5 Institute of Life Sciences, Heilongjiang Bayi Agricultural University, Daqing 163319, China

**Keywords:** cold region, rice cultivars, N fertilization strategies, photosynthetic characteristics, dry material production

## Abstract

Nitrogen (N) fertilizer regulates leaf photosynthesis and dry matter production in rice, and extensive genotypic differences in N responsiveness exist among cold-region japonica rice cultivars. A two-year field experiment was conducted in the city of Jiamusi, Heilongjiang Province, during 2023–2024, using two representative japonica cultivars: the small-panicle cultivar Longjing 47 (LJ47) and the large-panicle cultivar Longjing 3010 (LJ3010). A split-plot design was adopted with four N application rates (0, 110, 138, and 166 kg N·ha^−1^) and four basal–tiller to panicle–grain N ratios (10:0, 8:2, 7:3, and 6:4). The results showed that increasing N supply significantly enhanced SPAD values, photosynthetic capacity, leaf area index, and total dry matter accumulation in both cultivars. LJ47 maintained high photosynthetic capacity under moderate N supply, whereas LJ3010 required higher N input to sustain superior photosynthetic performance. From heading to maturity, dry matter accumulation, photosynthetic potential, crop growth rate, net assimilation rate, N uptake, and grain yield all increased with an increasing ratio of panicle–grain N fertilizer, with more pronounced improvements observed in LJ3010. LJ47 achieved optimal physiological performance and grain yield at 138 kg N·ha^−1^ (moderate N), with no further promotion under high-N supply. In contrast, LJ3010 required 166 kg N·ha^−1^ (high-N) to maintain photosynthetic advantages and achieve higher grain yield in the late growth stage. Increasing the proportion of basal–tiller N fertilizer favored the source sink balance of LJ47, while increasing panicle–grain topdressing delayed leaf senescence and promoted post-heading dry matter translocation in LJ3010. For a target grain yield of 9 t·ha^−1^, the recommended N rate is 138 kg·ha^−1^ with a basal–tiller to panicle–grain N ratio of 8:2–7:3 for LJ47, and 166 kg·ha^−1^ with a ratio of 7:3–6:4 for LJ3010. This study provides a theoretical basis and data reference for the formulation of precise N fertilization strategies that match fertilizer management with rice cultivar characteristics in cold regions.

## 1. Introduction

Rice is a major staple grain crop in Heilongjiang Province. In 2023, the rice-sown area reached 3.2685 million hectares, accounting for 21.36% of the province’s total crop-sown area and 11.29% of the rice planting area in China [1]. Rice yield potential is largely determined by photosynthetic carbon assimilation and subsequent assimilation partitioning to grains [2,3], with over 90% of rice grain yield being derived from leaf photosynthates [4]. N fertilizer serves as a critical regulator of rice photosynthetic capacity and dry matter accumulation; however, current rice production in cold regions is constrained by irrational N management practices, characterized by excessive total N input and unbalanced fertilizer proportions—specifically, overapplication of basal N and insufficient panicle N supply [5,6]. Such indiscriminate fertilization strategies fail to match the seasonal N demand of cold-region rice, leading to excessive vegetative growth, weakened cold and lodging resistance, and reduced N use efficiency [7]. Moreover, rice cultivars show significant genotypic variation in N uptake, utilization, and assimilate allocation [8,9,10,11]. Commercial rice cultivars in cold regions are currently divided mainly into large-panicle and small-panicle ecotypes, which differ markedly in photosynthetic performance [12,13]. Given the substantial genotypic differences in N responsiveness among rice cultivars, conventional uniform fertilization practices cannot fully exploit the photosynthetic and dry matter production potential of diverse rice varieties [14].

Numerous studies have demonstrated that, under optimal total N application, increasing the ratio of N topdressing can effectively improve the population photosynthetic rate of rice during the middle and late growth stages and can thus significantly enhance grain yield [15,16,17]. However, cold rice-growing regions are characterized by limited thermal resources and a short growing season, which require N management strategies that optimize early population establishment and maintain late-season physiological functions. Previous studies have focused primarily on single-cropping rice systems [18,19,20] or rice production in southern China [21,22,23]. Regarding northern cold-region rice production, Ji et al. [24] investigated the effects of controlled-release urea on N use efficiency but neglected panicle-type differences. Similarly, Qi et al. [25] compared N utilization among different rice genotypes without panicle-type classification, and Zheng et al. [26] analyzed provincial-scale N use efficiency in Heilongjiang but ignored variations among different panicle-type cultivars. The differential N responsiveness of rice cultivars is jointly determined by their inherent source–sink architecture, assimilate translocation characteristics, and the unique ecological conditions of cold regions. Overall, few studies have systematically compared N response discrepancies between large-panicle and small-panicle rice cultivars in cold regions by integrating aboveground physiological traits and belowground nutrient uptake characteristics [27,28]. Although our research group previously explored the effects of N fertilizer on grain yield and the yield components of cold-region rice cultivars with contrasting panicle types [29], dominant local rice cultivars have been updated in recent years. More importantly, the N adaptability of different panicle-type varieties has not been elucidated from the perspective of physiological source–sink–flow mechanisms. Therefore, the present study selects two widely cultivated and representative cold-region japonica rice cultivars—the small-panicle cultivar LJ47 and the large-panicle cultivar LJ3010—as experimental materials. Field experiments were conducted with gradient N application rates and varied ratios of basal–tiller fertilizer to panicle–grain fertilizer. The objectives of this study are to (1) clarify the regulatory effects of N management on photosynthetic characteristics and dry matter accumulation, and reveal the physiological differences in N response between the two panicle-type rice cultivars; (2) elucidate the effects of N fertilization on yield components and N use efficiency based on the genetic characteristics of different rice cultivars, and determine the optimal N application rate and fertilizer allocation pattern for large-panicle and small-panicle rice cultivars in cold regions; (3) provide a theoretical basis and technical support for high-yield and high-efficiency N management in cold-region rice production.

## 2. Results

Analysis of variance (ANOVA) showed that the main effects of cultivar, N application rate, and N application ratio were highly significant, whereas the effect of year was non-significant. The interactions between the variables cultivar and N application rate, as well as between cultivar and N application ratio, reached a highly significant level. Meanwhile, the three-way interaction of cultivar × N application rate × N application ratio was also highly significant (see Table 1).

**Table 1:** Analysis of variance of N rates and N ratios on grain yield and main morphophysiological indicators of the rice cultivars.

Source of Variation	Degree of Freedom	Grain Yield	Pn	SPAD	LAI	Dry Matter	Grain Numbers per Ear	N Absorption
Year(A)	1	1.14 ns	34.10 **	0.41 ns	55.08 ns	17.31 ns	1.61 ns	2.89 ns
Variety(B)	1	614.86 **	768.55 **	61.38 **	1621.86 **	2884.49 **	14281.82 **	1414.67 **
N rate(C)	3	859.42 **	1036.08 **	93.47 **	36,110.16 **	9827.25 **	4344.94 **	5050.90 **
Ratio (D)	3	98.66 **	74.14 **	20.61 **	260.25 **	72.43 **	75.99 **	57.65 **
A × B	1	114.01 **	0.61 ns	0.47 ns	55.82 **	3.68 ns	51.29 **	6.11 *
A × C	3	2.62 ns	11.07 **	4.80 *	33.77 **	142.94 **	18.47 **	0.57 ns
A × D	3	24.36 **	2.04 ns	0.89 ns	8.47 **	2.22 ns	0.65 ns	0.95 ns
B × C	3	41.48 **	38.87 **	58.36 **	677.72 **	123.48 **	30.55 **	64.12 **
B × D	3	4.97 **	70.52 **	11.84 **	174.14 **	37.22 **	22.92 **	34.32 **
C × D	9	13.84 **	8.97 **	2.84 *	25.97 **	13.24 **	8.03 **	5.58 **
A × B × C	3	78.74 **	5.81 **	0.78 ns	30.67 **	87.11 **	4.08 **	7.04 **
A × B × D	3	6.75 **	2.17 ns	1.48 ns	20.06 **	2.09 ns	2.62 **	0.34 ns
A × C × D	9	6.47 **	0.38 ns	3.60 **	4.03 **	3.58 **	1.69 ns	0.52 ns
B × C × D	9	5.05 **	5.24 **	1.55 ns	14.82 **	13.40 **	4.73 **	5.44 **
A × B × C × D	9	2.27 *	0.65 ns	0.80 ns	4.33 **	4.29 **	2.34 *	1.08 ns
Whole-plot error	12	—	—	—	—	—	—	—
Subplot error	112	—	—	—	—	—	—	—

Note: The data presented in the table correspond to F-values for each tested effect. ** and * denote significance at *p* < 0.01 and *p* < 0.05, respectively, while “ns” indicates non-significant differences. F-values were calculated using Type III sums of squares with Satterthwaite’s approximation for denominator degrees of freedom. The whole-plot error (df = 12) serves as the error term for Year, N rate, and their interaction; the subplot error (df = 112) serves as the error term for Variety, Ratio, and all other interactions. The denominator df are consistent with the split-plot design comprising 2 years × 3 blocks × 4 N rates (main plots) and 2 varieties × 4 N ratios (subplots), totaling 192 observations.

### 2.1. The Effects of N Application on the Photosynthetic Characteristics of Different Rice Cultivars

#### 2.1.1. N Application Rate

**Figure 1:**
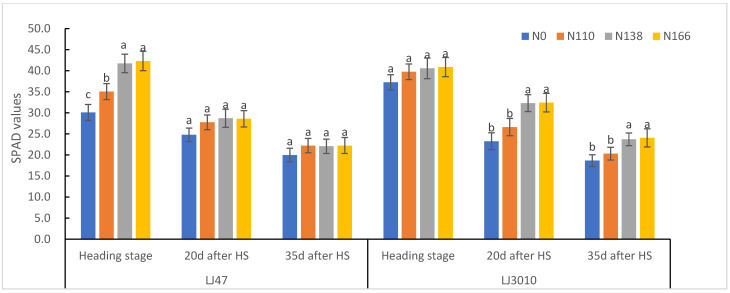
The effects of different N application rates on leaf SPAD values of two rice cultivars in 2023 and 2024. Note: Since similar data trends were observed in 2023 and 2024, the two-year average values are shown in this figure. Different little letters indicate significant differences at the 0.05 level under different treatments (*p* < 0.05); the same as follows.

The N application rate significantly influenced the leaf SPAD values of the two rice cultivars after heading (Figure 1). The SPAD values of the top three functional leaves of both cultivars increased gradually with rising N input. In the small-panicle cultivar LJ47, SPAD values showed no significant difference between the medium-N (N 138 kg·ha^−1^) and high-N (N 166 kg·ha^−1^) treatments; these two treatments had markedly higher SPAD values than the low-N (N 110 kg·ha^−1^) and zero-N treatments. As rice developed after heading, the discrepancies among N treatments gradually narrowed, and no significant differences were observed at 20 and 35 days after heading. In the large-panicle cultivar LJ3010, no significant inter-treatment difference in the SPAD values of the top three functional leaves was detected at the heading stage. At 20 and 35 days after heading, the medium- and high-N treatments had similar SPAD values, both of which were significantly higher than the values produced by the low-N and zero-N treatments.

The N application rate significantly affected the photosynthetic characteristics of the two rice cultivars (Table 2). Net photosynthetic rate (Pn), stomatal conductance (Gs), and transpiration rate (Tr) increased with elevated N supply, whereas intercellular CO_2_ concentration (Ci) decreased. At the heading and maturity stages, the Pn, Gs, and Tr of the small-panicle cultivar exhibited no obvious differences between the medium- and high-N treatments, but were markedly higher than those under the low- and no-N treatments. In the large-panicle cultivar, these photosynthetic parameters were significantly greater under high-N conditions than under medium-, low-, or no-N treatments. These results indicated that the small-panicle cultivar can maintain high photosynthetic capacity under moderate N input, while the large-panicle cultivar requires higher N application to sustain superior photosynthetic performance.

The photosynthetic characteristics of the two rice cultivars were significantly affected by the N application rate (Table 2). Net photosynthetic rate (Pn), stomatal conductance (Gs), and transpiration rate (Tr) increased with rising N supply, while intercellular CO_2_ concentration (Ci) decreased correspondingly. At the heading and maturity stages, no significant differences in Pn, Gs, and Tr were detected between the medium-N and high-N treatments applied to the small-panicle cultivar, though these two treatments yielded remarkably higher values than the low-N and zero-N treatments. By contrast, all the above photosynthetic parameters were significantly higher under the high-N treatment than under the medium-N, low-N, and zero-N treatments applied to the large-panicle cultivar. These findings revealed that the small-panicle cultivar was capable of maintaining strong photosynthetic capacity under moderate N input, whereas the large-panicle cultivar required a higher N application rate to sustain superior photosynthetic performance.

#### 2.1.2. N Application Ratio

**Figure 2:**
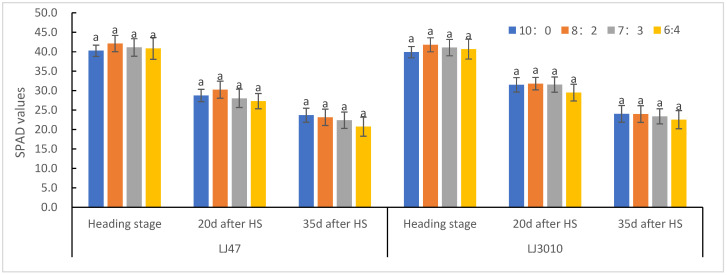
The effects of different N application ratios on SPAD values of two rice cultivars in 2023 and 2024.

The post-heading SPAD values of the two rice cultivars were not significantly influenced by the N application ratios of early-stage (basal and tillering fertilizer) and late-stage (panicle and grain fertilizer) N (Figure 2). However, the photosynthetic parameters of both cultivars were markedly affected by these N application ratios (Table 3). Regarding the small-panicle cultivar, significantly higher photosynthetic capacity was observed at the heading stage in the 8:2 ratio treatment than in the 10:0 and 6:4 treatments, while no significant difference was detected relative to the 7:3 treatment. At the maturity stage, significantly lower photosynthetic capacity was exhibited by the 10:0 treatment in comparison with all other treatments. In contrast, the large-panicle cultivar exhibited higher photosynthetic capacity in the 7:3 and 6:4 treatments at both the heading and maturity stages, and significantly better performance was achieved by these two treatments relative to the 10:0 and 8:2 treatments. These findings indicate that photosynthetic performance in small-panicle rice cultivars benefits from increases in the proportion of early-stage N fertilizer, whereas the high photosynthetic performance of large-panicle cultivars can be maintained by increasing late-stage topdressing.

### 2.2. The Effects of N Application Rates on the Leaf Area Index of Different Rice Cultivars

#### 2.2.1. N Application Rate

**Figure 3:**
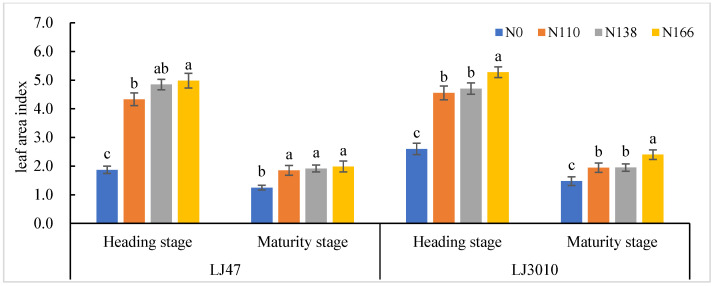
The effects of different N rates on leaf area index of two rice cultivars in 2023 and 2024.

The N application rate significantly affected the leaf area index (LAI) of the two rice cultivars at heading and maturity stages (Figure 3). In both cultivars, LAI increased markedly with increasing N supply at the heading stage. Across the two experimental years, no significant differences in LAI were detected between high-N and medium-N treatments at the heading and maturity stages of the small-panicle cultivar, with both treatments producing significantly larger LAI values relative to the low-N and zero-N (N0) treatments. In the large-panicle cultivar, the two-year average LAI under the high-N treatment at the heading and maturity stages was 1.18 times that of the medium-N treatment. Collectively, LAI under the high-N treatment was significantly higher than under the medium-N, low-N, and zero-N treatments.

#### 2.2.2. N Application Ratio

**Figure 4:**
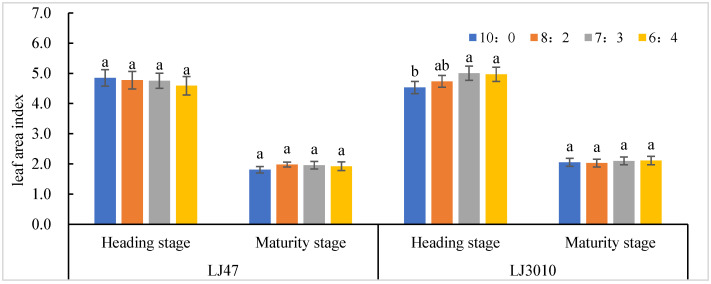
The effects of different N ratios on leaf area index of two rice cultivars in 2023 and 2024.

The N application ratio of basal–tillering fertilizer to panicle–grain fertilizer significantly affected the leaf area index (LAI) of the two rice cultivars at the heading and maturity stages (Figure 4). In the small-panicle cultivar, no significant differences in LAI were detected among different fertilization treatments at both growth stages in 2023 and 2024. In the large-panicle cultivar, the 7:3 and 6:4 basal–tillering to panicle–grain N ratios yielded significantly higher LAI values than the 10:0 ratio at the heading stage. Additionally, no significant differences in LAI were observed among all treatments at the maturity stage across the two experimental years. These results indicated that reducing the proportion of basal and tillering N fertilizer while increasing panicle–grain N input is an effective approach to improving the LAI of large-panicle rice cultivars.

### 2.3. The Effects of N Fertilizer on the Growth Indicators of Different Rice Varieties at the Heading Stage

#### 2.3.1. N Application Rate

The N application rate significantly altered rice population growth indicators at the heading stage (Table 4). Dry matter accumulation, single-stem leaf area, leaf weight, and efficient leaf area ratio increased with elevated N supply. In the small-panicle cultivar, no significant differences in these four indicators were found between the medium- and high-N treatments across 2023 and 2024. In comparison, significant differences were observed in all indicators of the large-panicle cultivar, except for single-stem leaf area, between the two N treatments over the two experimental years. Two-year average results showed that high-N treatment increased dry matter accumulation by 56.77 g·m^−2^, leaf weight by 15.59 g·m^−2^, and efficient leaf area ratio by 2.93%, relative to the medium-N treatment at the heading stage.

#### 2.3.2. N Application Ratio

N application ratios between the early and late growth stages significantly modulated population dry matter accumulation and leaf area composition at the heading stage. In the small-panicle cultivar, heading-stage dry matter accumulation and leaf weight declined with the increasing proportion of late-stage N fertilizer over the two experimental years. Specifically, the 10:0 and 8:2 treatments yielded greater dry matter accumulation and leaf weight than the 7:3 and 6:4 treatments, with the 8:2 treatment showing significantly higher values than the 6:4 treatment. No significant differences in single-stem leaf area and efficient leaf area ratio were observed among the different N ratio treatments applied to the small-panicle cultivar. In the large-panicle cultivar, heading-stage dry matter accumulation increased as the proportion of late-stage N application rose. In 2023, the 7:3 and 6:4 treatments exhibited significantly higher leaf weight, single-stem leaf area, and efficient leaf area ratio than the 10:0 treatment. In 2024, these three indicators were significantly greater under the 7:3 treatment relative to the 10:0 treatment. These results demonstrate that increasing the proportion of panicle and grain fertilizer reduced heading-stage dry matter accumulation and leaf weight in the small-panicle cultivar, while improving these growth parameters in the large-panicle cultivar (Table 5).

### 2.4. The Effects of N Fertilizer on Photosynthetic Matter Production and Accumulation in Two Rice Cultivars After Heading

#### 2.4.1. N Application Rate

Different N fertilizer rates significantly affected the photosynthetic dry matter production of rice populations during the late growth stage (from heading to maturity). In both rice cultivars, N application treatments exhibited substantially higher net dry matter accumulation, grain yield, photosynthetic potential, population growth rate, and net assimilation rate compared with the zero-N treatment. The high-N treatment (166 kg·ha^−1^) achieved the maximum values of net dry matter accumulation, photosynthetic potential, and population growth rate. Grain mass per unit leaf area showed no significant differences among the high-, medium-, and low-N treatments, whereas all N treatments yielded significantly higher values than the zero-N group. In the small-panicle cultivar, no significant differences in net dry matter accumulation, photosynthetic potential, population growth rate, net assimilation rate, grain weight per leaf area, and grain yield were observed between the high- and medium-N treatments. In contrast, most of these indicators differed significantly between the two N levels as applied to the large-panicle cultivar (Table 6). Across the two experimental years, relative to the medium-N treatment, the high-N treatment increased net dry matter accumulation by 117.10 kg·ha^−1^, photosynthetic potential by 211.98 kg·ha^−1^, and grain yield by 20.03 kg·ha^−1^ on average during the heading-to-maturity stage. Additionally, the population growth rate was increased by 3.32 g·m^−2^·d^−1^ under high-N conditions in 2024. These findings indicate that the large-panicle cultivar had a higher N demand than the small-panicle cultivar under identical cultivation conditions, and increasing late-stage N application was effective in promoting population dry matter accumulation during the heading-to-maturity period. Across all N application treatments, the ratio of pre-heading dry matter accumulation to total dry matter accumulation was approximately 50%, while this ratio was lower than 50% in the zero-N treatment (Table 6). Further analysis demonstrated that, in the large-panicle cultivar, the high-N treatment balanced the contribution of pre- and post-heading dry matter accumulation to total dry matter, with post-heading dry matter accumulation serving as the dominant contributor to grain yield.

#### 2.4.2. N Application Ratio

Different N allocation ratios between early-stage (basal and tillering fertilizer) and late-stage (panicle and grain fertilizer) N application also significantly regulated population photosynthate production during the heading-to-maturity stage (Table 7). The 6:4 and 7:3 N treatments exhibited higher values of net dry matter accumulation, photosynthetic potential, population growth rate, net assimilation rate, and grain mass per unit leaf area. In the small-panicle cultivar, grain yield did not differ significantly between the 8:2 and 7:3 treatments, and both treatments produced considerably higher grain yields than the 10:0 treatment. In the large-panicle cultivar, no significant yield difference was detected between the 7:3 and 6:4 treatments, with both ratios yielding markedly higher values relative to the 10:0 treatment. Compared with the 10:0 N ratio, the 7:3 and 6:4 treatments increased the net dry matter accumulation of the small-panicle cultivar by an average of 38.82 kg·ha^−1^ and 54.07 kg·ha^−1^, and elevated grain yield by 736.7 kg·ha^−1^ and 674.7 kg·ha^−1^, respectively. The large-panicle cultivar showed greater improvement under adjusted N allocation; under the 7:3 and 6:4 treatments, respectively, its net dry matter accumulation increased by 95.22 kg·ha^−1^ and 149.33 kg·ha^−1^, while its grain yield increased by 841.9 kg·ha^−1^ and 713.2 kg·ha^−1^. These results demonstrate that increasing the proportion of late-stage N application conferred greater dry matter accumulation advantages in the large-panicle cultivar than in the small-panicle cultivar, ultimately contributing to the significantly higher grain yield of the former.

### 2.5. Effects of N Application on Rice Yield Components and N Uptake

#### 2.5.1. N Application Rate

Different N fertilizer rates significantly affected the yield components of rice cultivars. With increasing N input, 1000-grain weight remained relatively stable, while the number of effective panicles and grains per panicle varied substantially (Table 8). Compared with the high-N treatment, the medium-N treatment significantly increased the seed-setting rate (by 1.9%; *p* < 0.05) and improved N use efficiency by 4.4 percentage points, with other agronomic indicators exhibiting no significant differences between the two treatments. In contrast, in the large-panicle rice cultivars, high-N supply markedly elevated the number of grains per panicle relative to medium-N supply, whereas no significant differences were observed in seed-setting rate and N use efficiency. Under medium-N fertilization, the total N accumulation of small-panicle rice at harvest was 137.1 kg·ha^−1^ in 2023 and 140.2 kg·ha^−1^ in 2024, values that are consistent with the applied N rate of 138 kg·ha^−1^. These cultivars exhibited an average N requirement of 1.58 g per 100 kg of grain. In the large-panicle rice cultivars under high-N conditions, the total harvest N accumulation reached 165.1 kg·ha^−1^ in 2023 and 166.3 kg·ha^−1^ in 2024, closely matching the high-N application rate of 166 kg·ha^−1^, with an average grain N uptake of 1.79 g per 100 kg grain. These findings demonstrate that a medium-N rate is sufficient to support yield formation in small-panicle rice cultivars, whereas high-N input is indispensable to satisfying the nutrient demand and achieving optimal yield performance of large-panicle rice cultivars.

#### 2.5.2. N Application Ratio

N allocation ratios significantly altered rice yield components in this study. Variations in N partitioning had negligible effects on 1000-grain weight and panicle length. In small-panicle rice cultivars, the treatments with basal–tillering fertilizer to panicle–grain fertilizer ratios of 8:2 and 7:3 yielded higher effective panicle numbers, grains per panicle, and plant N uptake. Specifically, the 8:2 ratio substantially improved effective panicle number and plant N uptake relative to the 10:0 and 6:4 ratios, whereas the 7:3 ratio produced distinctly more grains per panicle than the 10:0 and 6:4 treatments. In large-panicle cultivars, no significant differences in effective panicle number, grains per panicle, or plant N uptake were observed between the 7:3 and 6:4 ratios; however, both ratios exhibited significantly superior performance compared with the 10:0 and 8:2 ratios (Table 9). Furthermore, the initial soil mineral N content in the 0–60 cm soil layer was 92.3 kg/ha. After the rice harvests in 2023 and 2024, the residual soil mineral N content for the LJ47 cultivar ranged from 87.4 to 93.6 kg/ha under the optimal N ratios of 8:2 and 7:3, while that of the LJ3010 cultivar ranged from 88.1 to 95.3 kg/ha under the optimal ratios of 7:3 and 6:4. Collectively, these findings suggest that small-panicle cultivars perform better under a higher proportion of basal and tillering fertilizer, whereas large-panicle cultivars require an increased proportion of panicle and grain fertilizer to achieve superior growth and yield.

## 3. Discussion

### 3.1. Regulation of Source–Sink–Flow System in Two Panicle-Type Rice Cultivars by N Application Rate

Nitrogen (N) fertilizer is a core factor that regulates leaf photosynthetic capacity, population structure, and dry matter translocation in rice and directly governs the source–sink balance of rice populations [30,31]. Nitrogen deficiency restricts leaf expansion, while excessive N application triggers excessive vegetative growth, dense canopy closure, and premature leaf senescence, which disturb the coordination between source and sink organs. The present study confirmed that increased N supply improved the chlorophyll content, leaf area index, leaf photosynthetic performance, and dry matter accumulation of both cold-region rice cultivars, outcomes that are in agreement with the results of previous studies [32,33]. There were significant genotypic differences in N responsiveness between the two tested cultivars. The small-panicle cultivar LJ47 achieved N saturation at the medium-N rate of 138 kg·ha^−1^. Further increasing N input to the high-N level of 166 kg·ha^−1^ resulted in no significant promoting effects on leaf area, dry matter accumulation, grain yield, or N use efficiency. Excessive N application failed to further enhance photosynthesis and N metabolism, and even decreased the seed-setting rate and N utilization efficiency [34,35]. LJ3010 needed a higher N input (166 kg·ha^−1^) to sustain stable leaf chlorophyll concentration and normal leaf gas exchange capacity during the late growth stage. Data pooled across two growing seasons showed that, relative to the medium-N treatment, the high-N application significantly increased net dry matter accumulation, photosynthetic potential, and grain yield by 117.10 kg·ha^−1^, 211.98 kg·ha^−1^, and 20.03 kg·ha^−1^, respectively. Total plant N uptake was generally proportional to the N application rate, further demonstrating the inherent differences in N demand between rice cultivars with contrasting panicle types.

### 3.2. Regulation of Source–Sink–Flow System in Two Panicle-Type Rice Cultivars by N Application Ratio

Rice yield formation depends on the coordinated function of sources (photosynthetic functional leaves), sinks (grain storage capacity), and flows (photosynthate translocation). The ratio of basal–tiller nitrogen fertilizer to panicle–grain nitrogen fertilizer determines seasonal N allocation patterns, thereby regulating the synergistic relationship among source, sink, and flow systems in rice [36,37]. Increasing the proportion of panicle–grain fertilizer at the late growth stage can effectively delay leaf senescence, prolong the photosynthetic duration, and promote post-heading photosynthate production in both cultivars, which is consistent with previous studies [38,39]. However, the present study further revealed an inherently divergent physiological regulatory mechanism of source–sink–flow coordination in small-panicle and large-panicle cold-region japonica rice, which has rarely been systematically quantified and clarified in existing regional rice fertilization research. The two cultivars displayed entirely distinct responses to nitrogen fertilizer ratios, which stemmed from their divergent yield formation pathways and canopy construction strategies.

For the small-panicle cultivar LJ47, early-stage basal and tillering N acts as the core driver to construct sufficient photosynthetic source populations. Sufficient pre-anthesis N supply promotes rapid tiller emergence, expands early leaf area index, and elevates the proportion of high-efficiency functional leaves at heading, establishing a stable “source foundation” to support subsequent grain filling. The results of the present study indicate that reducing the proportion of basal–tiller fertilizer while increasing panicle–grain fertilizer (the 6:4 treatment) significantly decreased leaf area and photosynthetic capacity at the heading stage. This phenomenon was evidenced by a reduced net photosynthetic rate (Pn), stomatal conductance (Gs), and transpiration rate (Tr), as well as elevated intercellular CO_2_ concentration (Ci) (Table 4). Late-stage N supplementation could not compensate for the insufficient canopy source supply, ultimately resulting in reduced dry matter accumulation, inhibited photosynthate translocation, decreased grain number per panicle, and limited sink capacity. In comparison, the treatment with full basal–tiller N application (the 10:0 treatment) lacked nitrogen input during the late growth stage, which led to reductions in effective panicle number, grain number per panicle, and seed-setting rate. Collectively, the balanced 8:2–7:3 N allocation ratio maintains a matching relationship between the source size and sink demand of LJ47, balancing early population construction and late leaf physiological activity. Unlike LJ47, the source–sink coordination of the large-panicle cultivar LJ3010 is mainly restricted by late-stage leaf senescence and the assimilate transport barrier rather than insufficient early leaf area. Our results demonstrate that increasing the proportion of panicle–grain fertilizer can maintain leaf photosynthetic performance, while facilitating the translocation of photosynthates and nitrogen to grains, thereby improving dry matter accumulation and total plant N uptake. This optimized fertilization regime unblocked assimilate transport and satisfied the vigorous sink demand of large-panicle rice cultivars [40]. The grain–leaf ratio, a core indicator for evaluating source–sink coordination, was significantly improved by postponed N application at the late growth stage, with more prominent effects observed in the large-panicle cultivar [41]. A high proportion of basal–tiller N (10:0, 8:2) leads to redundant vegetative growth, accelerates leaf senescence after heading, blocks the flow of photosynthates to grains, and fails to give full play to its yield potential of more grains per panicle. Thus, the recommended ratio of basal–tiller fertilizer to panicle–grain fertilizer for this cultivar ranges from 7:3 to 6:4.

Such genotype-specific regulatory differences constitute the core physiological basis for differentiated N management. Most earlier studies adopted unified N allocation schemes regardless of panicle morphotypes. This study clarified two unique physiological pathways: small-panicle cultivars expand source scales via early basal N, while large-panicle cultivars depend on late topdressing to facilitate assimilate translocation. The mechanistic findings enrich the theoretical system of precise N management for cold-region rice and provide solid support for our cultivar-targeted fertilization recommendations.

### 3.3. Regional Characteristics of N Responses of Different Rice Cultivars and Rational N Fertilizer Recommendations

How responsive rice is to N is tightly regulated by climatic conditions [42], cultivar genotype [43], and soil properties [44]. In southern rice-producing regions, high early-season temperatures accelerate soil nutrient mineralization. Rice plants in these regions are predominantly sensitive to N during the mid-to-late grain-filling stages and exhibit a relatively narrow N tolerance range [45]. In contrast, low temperatures during the early growing season in cold regions inhibit soil N supply. Physiologically, differences in canopy structure, photosynthetic characteristics, and N translocation capacity collectively contribute to the divergent N responses of the two tested cultivars. Specifically, the LJ47 cultivar requires sufficient N mainly at the tillering stage, whereas LJ3010 maintains a high-N demand throughout the late grain-filling period. Japonica rice cultivars cultivated in southern and northern China exhibit distinct differences in total N demand. For example, japonica rice grown in Jiangsu Province and cold-region japonica rice take up approximately 2.0 kg and 1.4 kg of N per 100 kg of grain yield, respectively [46,47]. To achieve the same grain yield level, cold-region rice accumulates less total N than rice cultivated in Jiangsu Province, which is a primary cause of the divergent N application rates of these two rice production regions [48].

Ju et al. [49] and Peng et al. [50] reported that nitrogen loss from paddy fields in Heilongjiang Province is approximately equivalent to the N input from non-synthetic fertilizer sources, suggesting that the theoretical N application rate is consistent with the total N uptake removed by rice plants at harvest. This N fertilization recommendation framework for cold-region paddy fields has been validated in our previous studies [51]. Accordingly, based on analyses of photosynthetic performance, dry matter accumulation and yield components, combined with total plant N uptake and N use efficiency, and under the target grain yield of 9 t·ha^−1^ for local cold-region paddy fields, the optimal N application rate for LJ47 is recommended as 138 kg·ha^−1^, with a basal–tiller to panicle–grain fertilizer ratio of 8:2 or 7:3.The recommended N rate for the large-panicle cultivar LJ3010 is 166 kg·ha^−1^, with a basal–tiller to panicle–grain fertilizer ratio of 7:3 or 6:4. The interaction effects of N rate and ratio on rice yield also confirmed this conclusion. When this optimized N management regime is adopted for both cultivars, soil mineral N concentration after harvest remains nearly consistent with the initial pre-transplanting level (Table 9). This finding further demonstrates that the optimized fertilization strategy balances N input and output in cold-region rice ecosystems, effectively mitigating production risks. Specifically, insufficient N supply weakens canopy growth, while excessive N input reduces cold tolerance and lodging resistance in rice plants [52]. Notably, the nitrogen fertilizer application rates proposed in this study were determined based on plant nitrogen demand and soil nitrogen balance, without monitoring nitrogen leaching and runoff. The main reason was that N leaching and runoff losses are minimal in cold-region paddy fields. According to in situ field measurements conducted by Bi et al., when nitrogen application rates in cold-region paddy fields range from 75 to 165 kg/ha, nitrogen leaching losses and nitrogen runoff losses account for 0.25–2.38% and 1.01–1.06% of the applied nitrogen, respectively [53]. From the perspective of source–sink–flow theory, this study quantified the N uptake coefficients as 1.58 kg and 1.79 kg per 100 kg grain yield for LJ47 and LJ3010, respectively. In practical production, the regional N application rates for the two cultivars can be flexibly adjusted based on the cultivar-specific N uptake coefficients determined in this study and the local target grain yield, so as to achieve precise and cultivar-matched N fertilization management for cold-region rice production.

## 4. Materials and Methods

### 4.1. Experimental Site

Field experiments were carried out in 2023 and 2024, with the 2024 experiment built upon the experimental arrangement of 2023. The trials were conducted at the Rice Research Institute, Heilongjiang Academy of Agricultural Sciences, Jiamusi, Heilongjiang Province, China (46°52′ N, 130°20′ E). This region features a cold temperate continental monsoon climate, with an average altitude of 100 m, an annual mean temperature of 3.0 °C, an annual average precipitation of 496.7 mm, and an average frost-free period of 134 days. The experimental soil type is black soil.

### 4.2. Experimental Design

Four N (N) application rates were arranged in this study: N0 (zero-N application), N1 (20% reduction in local conventional N rate, 110 kg pure N·ha^−1^), N2 (local conventional N application rate for rice production, 138 kg pure N·ha^−1^), and N3 (20% increase in local conventional N rate, 166 kg pure N·ha^−1^). Total N fertilizer was split into four applications: basal fertilizer, tillering fertilizer (applied at the 4-leaf stage), panicle fertilizer (applied at the penultimate 4-leaf stage), and grain-filling fertilizer (applied at the penultimate 2-leaf stage). Four allocation ratios of basal plus tillering fertilizer to panicle plus grain-filling fertilizer were set, i.e., 10:0, 8:2, 7:3, and 6:4 (Table 10). All phosphorus fertilizer was applied entirely as basal fertilizer in a single application. For potassium fertilizer, 50% was applied as basal fertilizer and the remaining 50% as panicle fertilizer. N fertilizer was urea (46% N), phosphorus fertilizer was calcium superphosphate (46% P_2_O_5_), and potassium fertilizer was potassium sulfate (50% K_2_O). The field experiment was arranged in a split-plot design, with N application rates as the main plots and the ratio of basal–tillering fertilizer to panicle–grain fertilizer as the subplots. Each treatment was replicated three times in a randomized complete block design, resulting in 48 plots per year per variety. Across the two varieties, there were a total of 96 plots per year. The area of each experimental plot was 39 m^2^. Rice seeds were sown on April 20 via greenhouse dry nursery seedling raising, followed by manual transplanting. Uniform strong seedlings with a leaf age of 3.1–3.5 leaves were transplanted on May 20 at a spacing of 30 cm × 13.3 cm, with three seedlings per hill. The experimental field had medium soil fertility. The contents of soil organic matter, alkali-hydrolyzable N, available phosphorus, and available potassium were 30.1 g·kg^−1^, 121.5 mg·kg^−1^, 12.6 mg·kg^−1^, and 167.2 mg·kg^−1^, respectively, and the soil pH was 5.80. The initial mineral N content in the 0–60 cm soil layer was 92.3 kg·ha^−1^. Intermittent irrigation and field sun-drying practices were adopted throughout the growing season. Pest, disease, and weed management were consistent with local conventional rice cultivation practices. Plants were manually harvested per plot on October 1 for yield determination.

**Table 10:** The experiment design about the application of N fertilizer under two rice cultivars.

Treatments	N Rate (kg·ha^−1^)	N Ratio	Application Stage			
			Base Fertilizer (%)	Tillering Fertilizer (%)	Panicle Fertilizer (%)	Grain Fertilizer (%)
CK	0	0	0	0	0	0
T1	110	10:0	60	40	0	0
T2		8:2	48	32	12	8
T3		7:3	42	28	18	12
T4		6:4	36	24	24	16
T5	138	10:0	60	40	0	0
T6		8:2	48	32	12	8
T7		7:3	42	28	18	12
T8		6:4	36	24	24	16
T9	166	10:0	60	40	0	0
T10		8:2	48	32	12	8
T11		7:3	42	28	18	12
T12		6:4	36	24	24	16

### 4.3. Experimental Materials

(1) Rice Varieties: (1) LJ47 was jointly bred by the Jiamusi Rice Research Institute of Heilongjiang Academy of Agricultural Sciences, Heilongjiang Longke Seed Industry Group Co., Ltd., Harbin, China and Jiamusi Longjing Seed Industry Co., Ltd., Jiamusi, China. It was officially registered and approved by Heilongjiang Province in 2015 (Registration No. Hei Shen Dao 2015013). The whole growth duration is approximately 123 days, with a required active accumulated temperature ≥ 10 °C of around 2150 °C. This cultivar belongs to the small-panicle type and possesses strong tillering capacity. (2) LJ3010 is a promoted japonica rice cultivar adapted to the third thermal zone in cold regions of Heilongjiang Province. Developed by the Rice Research Institute of Heilongjiang Academy of Agricultural Sciences, it obtained provincial approval in 2021 (Registration No. Hei Shen Dao 2021L0105). Its growth period lasts roughly 127 days, requiring an active accumulated temperature ≥ 10 °C of approximately 2300 °C. This cultivar is characterized as a large-panicle type with moderate tillering ability.

(2) Fertilizers: N fertilizer: Urea (46% N); Phosphorus fertilizer: Diammonium phosphate (46% P_2_O_5_, 18% N); Potassium fertilizer: Potassium sulfate (50% K_2_O).

### 4.4. Measurement Items and Methods

(1) SPAD Value and Photosynthetic Characteristics: SPAD values were measured at the heading stage, 20 days after heading (DAH), and 35 (DAH). The relative chlorophyll content of the top three leaves of rice was determined with a SPAD-502 m. Three replicates were conducted for each treatment, which contained three biological replicates. Five consecutive hills were surveyed at each point, and one main stem was measured from each hill. The measurement was conducted in the order of flag leaf, penultimate leaf, and antepenultimate leaf. The measurement position was at the widest part of the leaf (approximately one-third of the leaf length from the leaf tip). Photosynthetic parameters of flag leaves were measured using a LI-6400 portable photosynthesis system (LI-COR Biosciences, USA at the heading stage and 35 DAH (maturity stage). Each measurement was repeated 4 to 6 times, and mean values were computed. The determined indices included net photosynthetic rate (Pn), stomatal conductance (Gs), transpiration rate (Tr), and intercellular CO_2_ concentration (Ci) of rice leaves.

(2) Dry Matter Accumulation and Leaf Area Measurement: Based on tiller population surveys at the heading stage, the average stem number per hill was calculated. Three representative plants were sampled per treatment at the full heading stage and maturity stage (a 40-day interval), with three biological replicates for each treatment. For each replicate, three uniform hills matching the plot average stem count were selected, and one medium-sized main stem was harvested from each hill to obtain three representative main stems per replicate. Subsequently, leaves were excised, and leaf length and width were sequentially measured for the flag leaf, penultimate leaf, and antepenultimate leaf. Leaves from the three sampled stems were individually packaged and oven-dried to quantify leaf dry weight. At the full heading stage, plant samples were divided into three components: leaf blades, panicles, and stem-sheaths. All samples were subjected to deactivation at 105 °C for 30 min and then dried to a constant weight at 80 °C. Notably, samples collected at the full heading stage and maturity stage were further separated into five components: green leaf blades, senesced leaf blades, panicles, stems, and sheaths. The leaf area of functional leaves was calculated using the length–width correction coefficient method.

(3) Biomass Yield, Yield Component and Rice Yield: Ten hills were sampled in each experimental plot to calculate the average number of effective panicles per hill. Afterwards, three hills with panicle numbers close to the plot average were chosen to determine the dry weight of panicles and straw. Total aboveground biomass production was defined as the sum of grain yield and straw biomass. Yield components: In each plot, 20 tillers were examined to determine the number of productive panicles. The average number of panicles per tiller was calculated, and based on this average, five representative tillers with typical plant height and panicle morphology were selected from distinct areas within the plot. Traits such as total grain number per panicle, filled grain number per panicle, and 1000-grain weight were measured for these samples. Each treatment was harvested separately to record the actual grain yield.

(4) Soil mineral N (NH_4_^+^-N and NO_3_^−^-N) was measured at transplanting (initial soil status) and maturity (post-harvest). Soil samples were collected in three layers: 0–20 cm, 20–40 cm and 40–60 cm. Cumulative mineral N stock in the 0–60 cm soil profile was calculated to support N balance analysis.

### 4.5. Parameter Calculation

(1) Leaf area (m^2^) = leaf length × maximum leaf width × correction coefficient; the correction coefficient is 0.75;

(2) Leaf area index (LAI) = total leaf area/corresponding land area;

(3) High-efficiency leaf area ratio (%) = high-efficiency LAI/maximum LAI × 100; where high-efficiency LAI refers to the flag leaf, penultimate leaf, and antepenultimate leaf of rice;

(4) Photosynthetic potential (×10^4^ m^2^·d·ha^−1^) = 1/2 (L1 + L2) (t2 − t1), where L1 and L2 are the leaf areas (m^2^·ha^−1)^ measured at the two consecutive times, and t1 and t2 are the times (d) of the two consecutive measurements;

(5) Net assimilation rate (g·m^−2^·d^−1^) = [ln (LAI2) − ln (LAI1)]/(LAI2 − LAI1) × (W2 − W1)/(t2 − t1), where LAI1 and LAI2 are the leaf area indices (m^2^·ha^−1^) measured in the two consecutive times, and t1 and t2 are the times (d) of the two consecutive measurements;

(6) Population growth rate (g·m^−2^·d^−1^) = (W2 − W1)/(t2 − t1), where W1 and W2 are the dry matter weights (t·ha^−1^) measured at the two consecutive times, and t1 and t2 are the times (d) of the two consecutive measurements;

(7) Grain weight/leaf area (mg·cm^−2^) = Grain yield/Leaf area at heading stage.

(8) N use efficiency (%) = (nutrient uptake of plants in the N-applied area − nutrient uptake in the no-N area)/amount of N applied × 100.

(9) The content of soil mineral N (kg·ha^−1^) = (NH_4_^+^-N and NO_3_^−^-N) × Soil depth × soil bulk density/10^5^.

### 4.6. Data Processing

Data sorting and statistical analysis were performed using Excel 2016 and SPSS 19.0. A four-factor split-plot analysis of variance (ANOVA) was adopted in this study. The N application rate was arranged as the whole-plot factor, whereas rice varieties and fertilizer ratio were set as subplot factors. Three-block repetitions nested within the trial year. Analysis of variance was performed using Type III sum of squares, and the Satterthwaite approximation was applied to correct denominator degrees of freedom for error term estimation. The least significant difference (LSD) method was applied for post hoc multiple comparisons, and statistical significance was determined at *p* < 0.05.

## 5. Conclusions

(1) Significant genotypic differences in N (N) demand existed between the two tested cold-region japonica rice cultivars. The small-panicle cultivar LJ47 performed best under moderate N supply, while the large-panicle cultivar LJ3010 required a high-N input to sustain superior growth and yield. Integrating photosynthetic traits, dry matter production, yield components, plant N uptake, and N use efficiency, the optimal N rates to reach a regional target yield of 9 t·ha^−1^ were identified as 138 kg·ha^−1^ for LJ47 and 166 kg·ha^−1^ for LJ3010.

(2) The two cultivars showed contrasting responses to N allocation ratios. A higher proportion of basal–tillering N (70–80%) improved photosynthesis, dry matter accumulation, and N uptake in LJ47. By contrast, increasing panicle–grain topdressing (60–70% basal–tillering N) maximized the productivity of LJ3010, a cultivar recommended for cold-region rice cultivation.

(3) Comprehensive physiological and agronomic indices can support the establishment of cultivar-specific precision N management regimes. The 100 kg grain N uptake coefficient derived from this study enables flexible adjustment of N schemes matching local target yields, which achieves both high grain yield and high-N use efficiency in cold-region rice.

## Figures and Tables

**Table 2:** The effects of different N application rates on photosynthetic characteristics of two rice cultivars at heading and maturity stages.

Varieties	N rate (kg·ha^−1^)	Heading Stage				Maturity Stage			
		Pn (µmol·m^−2^·S^−1^)	Gs (mol·m^−2^·S^−1^)	Ci (µmol·mol^−1^)	Tr (mmol·m^−2^·S^−1^)	Pn (µmol·m^−2^·S^−1^)	Gs (mol·m^−2^·S^−1^)	Ci (µmol·mol^−1^)	Tr (mmol·m^−2^·S^−1^)
LJ47	N0	16.5 c	0.31 c	280 a	4.1 c	7.0 c	0.14 c	304 a	1.6 c
	N110	18.1 b	0.46 b	260 b	5.2 b	8.3 b	0.22 b	280 b	2.4 b
	N138	23.5 a	0.59 a	222 c	6.9 a	12.0 a	0.31 a	242 c	3.2 a
	N166	23.1 a	0.62 a	229 c	6.6 a	12.1 a	0.29 ab	251 c	3.0 ab
LJ3010	N0	18.9 d	0.47 c	264 a	4.7 d	8.9 d	0.19 c	292 a	2.0 c
	N110	21.5 c	0.56 bc	251 b	6.2 c	10.4 c	0.24 bc	276 ab	2.6 bc
	N138	24.6 b	0.64 b	234 c	7.1 b	13.0 b	0.30 b	258 b	3.4 b
	N166	26.8 a	0.79 a	214 d	8.0 a	14.9 a	0.39 a	231 c	4.5 a

Note: Different little letters indicate significant differences at the 0.05 level under different treatments (*p* < 0.05); the same as follows.

**Table 3:** The effects of different N application ratios on photosynthetic characteristics of two rice cultivars at the heading and maturity stages.

Varieties	N Rate (kg·ha^−1^)	Heading Stage				Maturity Stage			
		Pn (µmol·m^−2^·S^−1^)	Gs (mol·m^−2^·S^−1^)	Ci (µmol·mol^−1^)	Tr (mmol·m^−2^·S^−1^)	Pn (µmol·m^−2^·S^−1^)	Gs (mol·m^−2^·S^−1^)	Ci (µmol·mol^−1^)	Tr (mmol·m^−2^·S^−1^)
LJ47	10:0	22.4 a	0.51 a	253 bc	6.1 ab	9.5 b	0.21 a	276 a	2.4 b
	8:2	22.7 a	0.53 a	249 c	6.5 a	10.7 a	0.25 a	274 a	2.6 ab
	7:3	21.3 ab	0.48 ab	265 ab	5.9 ab	11.3 a	0.27 a	268 a	2.9 a
	6:4	20.4 b	0.45 b	273 a	5.2 b	11.1 a	0.26 a	271 a	3.0 a
LJ3010	10:0	22.7 b	0.60 b	264 a	6.5 b	9.8 c	0.23 c	267 a	2.0 b
	8:2	23.4 b	0.63 ab	261 a	6.8 ab	11.2 b	0.28 b	256 b	2.5 b
	7:3	25.5 a	0.68 ab	232 c	7.2 a	12.5 a	0.33 a	238 c	3.3 a
	6:4	25.2 a	0.69 a	243 b	6.9 ab	12.1 a	0.35 a	241 c	3.6 a

**Table 4:** The effects of different N application rates on dry matter accumulation, components of population leaf area and grain–leaf ratio of two rice cultivars at the heading stage.

Year	Varieties	N Rate (kg·ha^−1^)	Dry Matter Accumulation (g·m^−2^)	Leaf Weight (g·m^−2^)	Single-Stem Leaf Area (cm^2^)	High-Efficiency Leaf Area Ratio (%)
2023	LJ47	N0	475.62 c	75.32 c	81.56 c	71.99 c
		N110	733.89 b	172.51 b	107.75 b	78.07 b
		N138	825.01 a	195.24 a	111.83 ab	83.40 a
		N166	820.66 a	195.97 a	116.07 a	82.20 ab
	LJ3010	N0	657.95 d	107.20 d	95.87 c	75.41 c
		N110	761.96 c	164.10 c	111.94 b	80.16 b
		N138	851.51 b	184.41 b	127.44 a	80.78 b
		N166	915.78 a	191.87 a	133.19 a	83.42 a
2024	LJ47	N0	424.56 c	75.32 c	80.30 c	71.88 c
		N110	770.48 b	181.24 b	106.50 b	77.29 b
		N138	813.55 a	195.49 a	110.83 ab	79.70 ab
		N166	817.21 a	198.73 a	116.64 a	82.90 a
	LJ3010	N0	698.84 d	105.27 c	96.94 c	76.63 c
		N110	823.98 c	182.62 b	117.68 b	76.24 c
		N138	884.01 b	187.16 b	127.08 ab	81.59 b
		N166	933.28 a	210.87 a	131.19 a	84.80 a

**Table 5:** The effects of different N application ratios on dry matter accumulation, components of population leaf area and grain–leaf ratio of two varieties at the heading stage.

Year	Varieties	N Ratio	Dry Matter Accumulation (g·m^−2^)	Leaf Weight (g·m^−2^)	Single-Stem Leaf Area (cm^−2^)	High-Efficiency Leaf Area Ratio (%)
2023	LJ47	10:0	796.01 ab	189.92 ab	109.18 a	82.54 a
		8:2	813.83 a	193.45 a	110.87 a	81.84 a
		7:3	786.22 b	187.35 ab	113.92 a	80.09 a
		6:4	783.35 b	180.90 b	113.54 a	80.43 a
	LJ3010	10:0	818.56 b	172.66 b	120.30 b	79.79 b
		8:2	825.17 ab	178.35 ab	123.93 ab	82.01 ab
		7:3	859.52 a	184.20 a	125.20 a	83.46 a
		6:4	869.07 a	185.29 a	127.32 a	83.21 a
2024	LJ47	10:0	834.00 a	205.00 a	110.04 a	79.99 a
		8:2	830.30 a	198.26 ab	112.49 a	82.24 a
		7:3	812.58 b	187.78 b	109.71 a	79.16 a
		6:4	716.45 c	162.90 c	113.06 a	79.13 a
	LJ3010	10:0	868.56 b	186.69 b	111.96 c	77.47 b
		8:2	879.50 a	185.35 b	121.26 b	80.40 b
		7:3	889.52 a	198.54 a	131.40 a	84.30 a
		6:4	884.10 a	203.63 a	136.66 a	81.32 ab

Note: N ratio means the proportion of N fertilizer application in early (base and tillering fertilizers) and late stages (panicle and grain fertilizers).

**Table 6:** The effects of N application rates on dry matter production in populations of two rice cultivars after heading stage.

Year	Varieties	N Rate (kg·ha^−1^)	Heading-Maturity				Photosynthetic Potential (×10^4^ m^2^·d ·ha^−1^)	Population Growth Rate (g·m^−2^·d^−1^)	Net Assimilation Rate (g·m^−2^·d^−1^)	Grain Weight/Leaf Area (mg·cm^−2^)
			Net Accumulation of Dry Matter (NA) (kg·ha^−1^)	Yield (kg·ha^−1^)	NA/Biomass (%)	NA/ Yield (%)				
2023	LJ47	N0	402.4 c	5011.0 c	45.83 b	80.31 b	60.00 c	10.06 b	6.83 c	19.92 b
		N110	789.46 b	7609.1 b	51.76 a	103.61 a	120.60 b	19.74 a	7.03 b	33.07 a
		N138	907.42 a	8737.3 a	52.37 a	103.91 a	128.15 a	22.69 a	7.56 a	33.94 a
		N166	911.52 a	8840.7 a	52.45 a	103.22 a	131.50 a	22.79 a	7.47 a	35.18 a
	LJ3010	N0	515.70 c	5248.4 c	43.94 b	98.26 b	70.40 c	12.89 c	6.62 c	19.53 b
		N110	864.93 b	8779.1 b	53.15 a	98.52 b	120.54 b	21.62 b	7.69 b	27.55 a
		N138	961.98 b	9223.1 ab	52.94 a	104.08 b	121.85 b	24.05 ab	8.41 a	31.86 a
		N166	1063.29 a	9320.5 a	53.70 a	114.00 a	140.34 a	26.58 a	8.05 a	31.42 a
2024	LJ47	N0	453.14 c	4822.2 c	53.46 a	93.97 a	50.70 c	8.20 b	6.60 c	17.78 b
		N110	756.14 b	8457.4 b	50.09 a	93.76 a	112.68 b	19.82 a	7.10 b	29.52 a
		N138	792.94 ab	8862.3 ab	47.98 a	85.32 a	117.92 ab	18.90 a	7.66 a	26.70 a
		N166	851.13 a	9035.1 a	52.14 a	94.20 a	126.98 a	22.28 a	7.66 a	27.30 a
	LJ3010	N0	474.81 c	5526.2 c	40.49 b	85.92 c	72.55 c	11.87 c	6.81 c	19.75 b
		N110	904.48 b	8679.1 b	50.40 a	104.21 b	120.28 b	22.61 b	7.62 b	26.81 a
		N138	867.91 b	8876.2 b	51.29 a	95.89 b	118.31 b	21.70 b	7.96 ab	26.52 a
		N166	1000.79 a	9202.8 a	51.58 a	111.16 a	139.88 a	25.02 a	8.09 a	23.80 a

**Table 7:** The effects of N application ratios on dry matter production in populations of two rice cultivars after heading.

Year	Varieties	N Ratio	Heading-Maturity				Photosynthetic Potential (×10^4^ m^2^·d·ha^−1^)	Population Growth Rate (g·m^−2^·d^−1^)	Net Assimilation Rate (g·m^−2^·d^−1^)	Grain Weight/Leaf Area (mg·cm^−2^)
			Net Accumulation of Dry Matter (NA) (kg·ha^−1^)	Yield (kg·ha^−1^)	NA/Biomass (%)	NA/ Yield (%)				
2023	LJ47	10:0	826.59 b	7956.2 b	50.84 a	103.70 a	125.40 a	20.66 a	7.11 b	33.48 a
		8:2	872.92 ab	8307.4 a	51.63 a	104.82 a	127.60 a	21.82 a	7.36 ab	34.52 a
		7:3	887.48 a	8603.4 a	53.13 a	103.63 a	126.73 a	22.27 a	7.52 a	34.10 a
		6:4	890.90 a	8715.8 a	53.17 a	102.16 a	127.27 a	22.19 a	7.43 a	34.16 a
	LJ3010	10:0	883.39 b	8734.8 b	51.88 a	101.02 b	118.20 b	22.08 b	7.99 ab	29.93 a
		8:2	933.02 b	8964.3 ab	53.07 a	103.97 ab	126.51 ab	23.33 ab	7.89 b	30.14 a
		7:3	1001.94 a	9382.3 a	53.77 a	106.63 ab	131.09 a	25.05 a	8.09 ab	30.43 a
		6:4	1035.24 a	9348.8 a	54.32 a	110.51 a	134.50 a	25.88 a	8.23 a	30.59 a
2024	LJ47	10:0	787.63 b	8190.2 c	47.53 a	96.17 a	114.06 b	19.69 a	6.72 b	26.82 b
		8:2	785.17 b	9019.9 a	49.08 a	87.05 a	116.69 ab	19.63 a	7.51 a	26.84 b
		7:3	804.37 ab	9016.4 a	52.49 a	89.21 a	122.47 a	20.11 a	7.61 a	28.36 a
		6:4	831.45 a	8780.0 b	51.18 a	94.70 a	123.58 a	21.91 a	8.04 a	29.33 a
	LJ3010	10:0	866.73 b	8312.9 b	49.79 a	104.26 a	117.57 c	21.67 b	7.03 b	24.01 b
		8:2	878.69 b	8856.9 ab	49.94 a	99.21 a	124.59 b	21.97 b	7.84 a	24.36 b
		7:3	938.61 b	9349.2 a	51.41 a	100.39 a	128.62 a	23.47 a	8.20 a	28.93 a
		6:4	1013.54 a	9125.2 a	53.22 a	112.30 a	133.74 a	25.34 a	8.49 a	26.54 ab

**Table 8:** The effects of different N rates on yield components and N absorption of two cultivars at maturity stage.

Year	Varieties	N Rate (kg·ha^−1^)	Panicle Number (Panicle·m^−2^)	Length of Ear (cm)	Grain Number per Ear (gain·ear^−1^)	Seed-Setting Rate (%)	1000-Grain Weight (g)	N Absorption (kg·ha^−1^)	N Fertilizer Use Efficiency (%)	Mineral N of 0–60 cm Soil Profile (kg·ha^−1^)
2023	LJ47	N0	422 c	13.1 b	53.4 c	97.4 a	26.5 a	85.6 c	—	62.2 c
		N110	447 b	14.2 a	74.2 b	95.5 b	26.3 a	130.8 b	41.1 a	82.6 b
		N138	476 a	14.6 a	80.5 a	94.5 b	26.2 a	137.1 ab	37.3 a	90.8 b
		N166	472 a	14.5 a	81.6 a	92.5 c	26.1 a	140.2 a	32.9 b	95.5 a
	LJ3010	N0	405 c	14.5 b	78.5 c	95.5 a	25.8 a	90.5 d	—	57.9 d
		N110	424 b	15.3 a	97.2 b	93.1 b	25.7 a	142.8 c	47.5 a	76.8 c
		N138	445 a	15.2 a	103.1 b	91.4 c	25.5 a	153.6 b	45.7 b	87.6 b
		N166	450 a	15.4 a	107.4 a	91.7 c	25.6 a	165.1 a	44.9 b	91.1 a
2024	LJ47	N0	413 c	13.6 b	50.3 c	96.9 a	26.1 a	83.8 c	—	56.9 d
		N110	445 b	14.6 a	76.7 b	95.8 ab	25.7 a	129.6 b	41.6 a	78.3 c
		N138	471 a	14.8 a	79.4 ab	94.7 b	25.8 a	140.2 ab	40.9 a	89.5 b
		N166	467 a	15.0 a	80.7 a	92.9 c	25.7 a	143.4 a	35.9 b	98.2 a
	LJ3010	N0	401 c	14.5 b	75.4 c	95.7 a	25.9 a	94.2 d	—	53.2 d
		N110	412 b	15.1 ab	100.5 b	93.6 b	25.5 a	147.8 c	48.7 a	70.4 c
		N138	432 a	15.3 a	105.3 b	92.6 b	25.6 a	155.2 b	44.2 b	83.7 b
		N166	436 a	15.5 a	111.4 a	92.7 b	25.6 a	166.3 a	43.4 b	89.3 a

**Table 9:** The effects of N application ratio on yield components and N absorption of two cultivars at maturity stage.

Year	Varieties	N Rate (kg·ha^−1^)	Panicle Number (Panicle·m^−2^)	Length of Ear (cm)	Grain Numbers per Ear (gain·ear^−1^)	Seed-Setting Rate (%)	1000-Grain Weight (g)	N Absorption (kg·ha^−1^)	Mineral N of 0–60 cm Soil Profile (kg·ha^−1^)
2023	LJ47	10:0	454 b	14.6 a	76.9 b	93.5 b	25.3 a	130.1 c	88.9 b
		8:2	465 a	14.5 a	78.4 ab	95.8 a	25.5 a	138.4 a	90.8 ab
		7:3	461 ab	14.7 a	81.5 a	96.5 a	25.5 a	136.8 ab	93.6 a
		6:4	457 b	14.5 a	77.8 b	96.1 a	25.7 a	131.4 bc	94.4 a
	LJ3010	10:0	424 a	15.1 a	100.5 b	90.6 b	25.3 a	150.8 b	85.6 b
		8:2	427 a	15.3 a	102.6 b	91.0 b	25.8 a	152.6 b	86.7 b
		7:3	428 a	15.4 a	105.3 a	94.2 a	26.1 a	164.5 a	92.2 a
		6:4	426 a	15.4 a	106.6 a	93.9 a	26.1 a	167.1 a	95.3 a
2024	LJ47	10:0	455 b	14.3 a	85.2 b	94.2 b	25.0 a	129.4 b	85.5 b
		8:2	464 a	14.5 a	87.1 a	96.2 a	25.4 a	136.4 a	87.4 ab
		7:3	460 a	14.7 a	88.3 a	96.7 a	25.4 a	138.4 a	89.6 a
		6:4	456 b	14.8 a	89.4 a	96.6 a	25.6 a	131.5 b	88.5 a
	LJ3010	10:0	415 a	15.3 a	101.6 b	91.2 b	25.8 a	145.3 b	79.6 b
		8:2	418 a	15.4 a	103.2 b	92.1 b	25.7 a	151.5 b	82.7 b
		7:3	417 a	15.6 a	107.1 a	94.7 a	26.0 a	166.1 a	88.2 a
		6:4	418 a	15.6 a	106.4 a	95.2 a	26.1 a	164.8 a	90.2 a

## Data Availability

The original contributions presented in this study are included in the article; further inquiries can be directed to the corresponding author.
